# Assessment of Ebola Virus Disease, Health Care Infrastructure, and Preparedness — Four Counties, Southeastern Liberia, August 2014

**Published:** 2014-10-10

**Authors:** Joseph D. Forrester, Satish K. Pillai, Karlyn D. Beer, Adam Bjork, John Neatherlin, Moses Massaquoi, Tolbert G. Nyenswah, Joel M. Montgomery, Kevin De Cock

**Affiliations:** 1 Epidemic Intelligence Service; 2 Division of Preparedness and Emerging Infections, National Center for Emerging and Zoonotic Infectious Disease; 3 Division of Global HIV/AIDS, Center for Global Health; 4 Division of Global Health Protection, Center for Global Health; 5 CDC Kenya, Center for Global Health, CDC; 6 Clinton Health Access Initiative; 7 Liberian Ministry of Health and Social Welfare

Ebola virus disease (Ebola) is a multisystem disease caused by a virus of the genus Ebolavirus (1,2). In late March 2014, Ebola cases were described in Liberia, with epicenters in Lofa County and later in Montserrado County (3). While information about case burden and health care infrastructure was available for the two epicenters, little information was available about remote counties in southeastern Liberia (Figure 1). Over 9 days, August 6–14, 2014, Ebola case burden, health care infrastructure, and emergency preparedness were assessed in collaboration with the Liberian Ministry of Health and Social Welfare in four counties in southeastern Liberia: Grand Gedeh, Grand Kru, River Gee, and Maryland. Data were collected by health care facility visits to three of the four county referral hospitals and by unstructured interviews with county and district health officials, hospital administrators, physicians, nurses, physician assistants, and health educators in all four counties. Local burial practices were discussed with county officials, but no direct observation of burial practices was conducted. Basic information about Ebola surveillance and epidemiology, case investigation, contact tracing, case management, and infection control was provided to local officials.

At the time of the evaluation, no cases of Ebola infection had been reported from any of the four counties. Each county has one referral hospital (100–150 beds) with outlying health centers and 17–24 clinics. Before the epidemic, six physicians served all four counties (range = one to three per county). At the time of the evaluation, only three physicians remained; the others had left Liberia because of the epidemic. In two of four hospitals assessed, nursing staff members were not coming to work or had abandoned facilities; in another hospital, health care providers had not been paid for 3 months but were still providing basic care. Frequently, nursing students, nursing aides, and community health care volunteers were providing basic medical care and responding to obstetric and surgical emergencies.

Supplies of nonsterile gloves and sterile obstetric and surgical gloves were depleted or absent in all four counties. Hand washing stations rarely were available in the facilities assessed, and if available, were typically located only in operating theaters. Hand washing stations in most health care settings consisted of water jugs, and even these were scarce. To compensate, bamboo hand washing stations were constructed for use at entrances to hospitals, county checkpoints, and in towns (Figure 2). Supplies of soap, bleach, or alcohol-based hand gel also were depleted. Rudimentary isolation facilities were present in two counties; neither had water, electricity, or waste disposal facilities. Communication between the county health office and hospitals and clinics relied on cell phones and radios, with intermittent Internet availability. In one county, only six of 19 health facilities had radio or cell phone contact with the health office; the other 13 required site visits by a district health officer. Transportation of specimens and patients was challenging; the counties each had only one functioning ambulance for all medical or specimen transfer, and no air transport was available.

Ebola emergency preparedness plans at the county and hospital level were lacking. Although Ebola task forces had been established in each county, according to reports from the field, the infrastructure and leadership were hampered by limited resources and difficulty communicating with and mobilizing the local communities. In all counties, there was insufficient personal protective equipment to care for patients with Ebola. Health care providers had not received training on the donning and removal of personal protective equipment. No training on case investigation, case management, contact tracing, or safe burial practices had been provided at either the county or hospital level. No Ebola surveillance systems were in place.

After basic training on case definitions and surveillance was provided to local officials, River Gee County health officials reviewed recent deaths and identified a patient with suspected Ebola. On August 3, a pregnant woman (patient 1) died during a spontaneous abortion after leaving Monrovia where she had contact with an infected person at a funeral; she was buried by the community in the week after her death. On August 24, 2014, Maryland County authorities identified a man hiding in a rice truck who had signs and symptoms of Ebola (patient 2). The truck had departed from Fish Town, River Gee County, and was destined for Pleebo, Maryland County. The man, who was reported to have participated in the burial of patient 1, was sent back to Fish Town, where he later was reported to have died of laboratory-confirmed Ebola. This was the first evidence of secondary transmission of Ebola in southeast Liberia.

Although additional Ebola cases have been reported in southeastern Liberia since this assessment was completed, there have been improvements in the level of Ebola preparedness. County health care staff received multiple trainings on surveillance, infection prevention and control practices, and burial practices. County Ebola task force meetings take place regularly, and an Ebola incident management system is in place. Additional ambulances and pickup trucks have been provided to county health teams. Three Ebola treatment units and multiple community care centers are planned for these southeastern counties. Still, obstacles to preventing spread of Ebola remain, and personal protective equipment,* sufficient personnel for effective contact tracing and case management,† efficient patient transport, and regional diagnostic laboratory capabilities are urgently needed. The Ebola disease case burden in southeastern Liberia is still lower than other areas of Liberia, but additional public health actions to strengthen preparedness and response efforts are needed to prevent further disease spread.

The latest updates, including case counts, on the 2014 Ebola outbreak in West Africa are available at http://www.cdc.gov/vhf/ebola/outbreaks/guinea/index.html. The most up-to-date clinical guidelines on the 2014 Ebola outbreak in West Africa are available at http://www.cdc.gov/vhf/ebola/hcp/index.html.

## Figures and Tables

**FIGURE 1:**
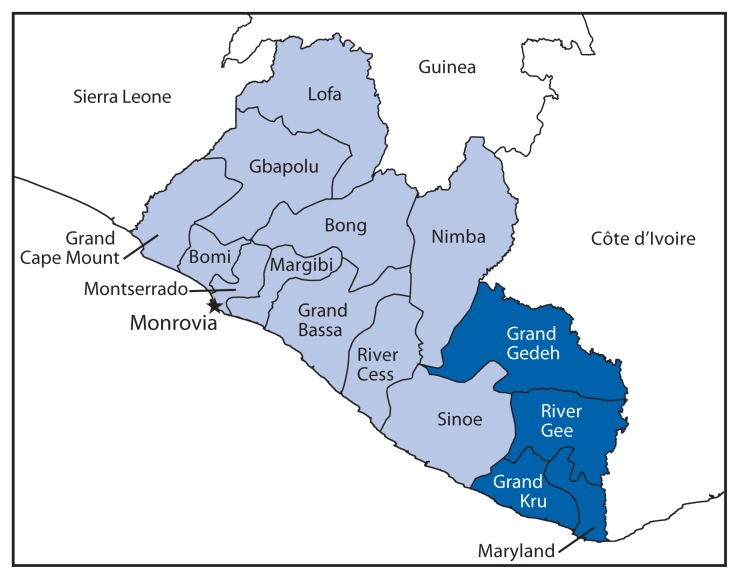
Location of the four counties assessed for Ebola virus disease case burden, health care infrastructure, and preparedness — Liberia, August 2014

**FIGURE 2:**
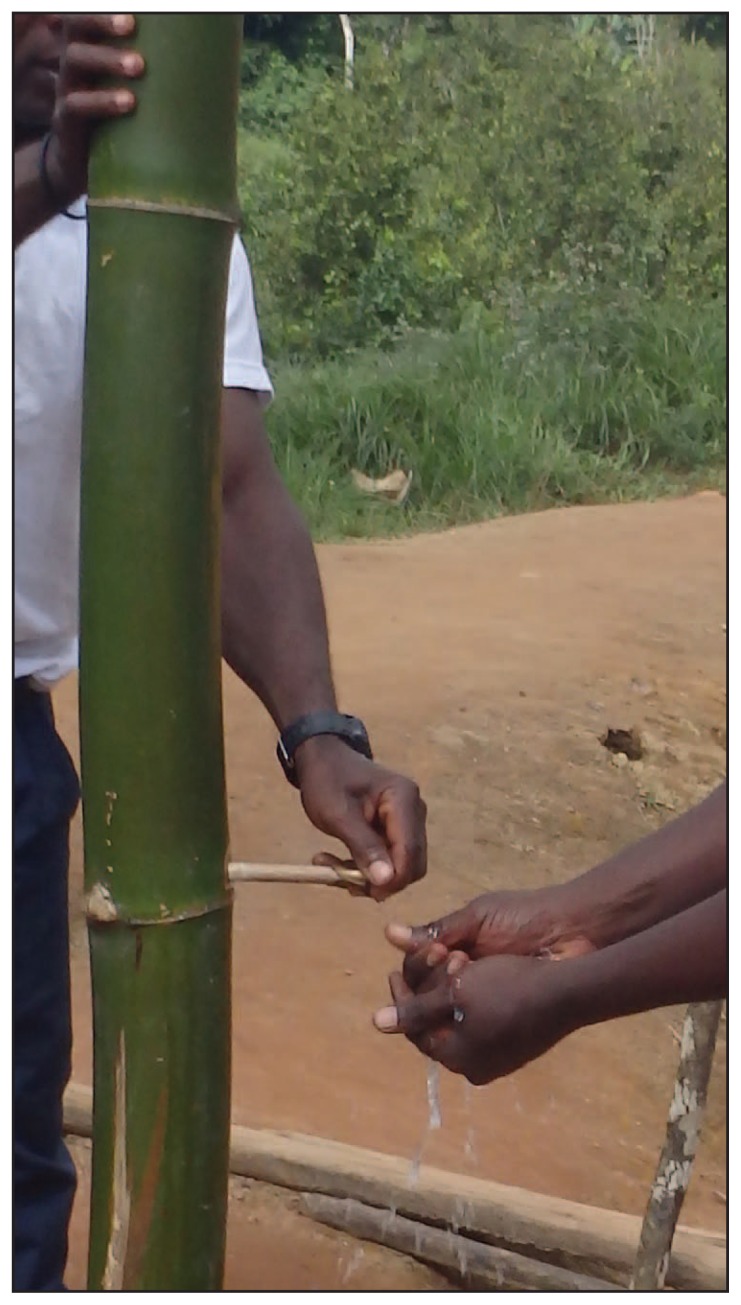
Residents use one of the bamboo hand washing stations* that were erected to improve health care practices at entrances to hospitals, county checkpoints, and in towns — Liberia, August 2014 * The diaphragms in the upper part of the bamboo stem are perforated to create a tube that can be filled with water. A hole is drilled just above the lowest intact diaphragm, then plugged with a small stick. The plug is removed to produce a stream of water.

## Footnotes

* Additional information available at http://www.cdc.gov/hai/prevent/ppe.html.

† Additional information available at http://www.cdc.gov/vhf/ebola/hcp/monitoring-and-movement-of-persons-with-exposure.html.
